# Vav1: A Dr. Jekyll and Mr. Hyde protein – good for the hematopoietic system, bad for cancer

**DOI:** 10.18632/oncotarget.5086

**Published:** 2015-08-20

**Authors:** Shulamit Katzav

**Affiliations:** ^1^ Developmental Biology and Cancer Research, IMRIC, Hebrew University-Hadassah Medical School, Jerusalem, Israel

**Keywords:** Vav1, Rac, GEF, RhoGTPases, Cancer

## Abstract

Many deregulated signal transducer proteins are involved in various cancers at numerous stages of tumor development. One of these, Vav1, is normally expressed exclusively in the hematopoietic system, where it functions as a specific GDP/GTP nucleotide exchange factor (GEF), strictly regulated by tyrosine phosphorylation. Vav was first identified in an NIH3T3 screen for oncogenes. Although the oncogenic form of Vav1 identified in the screen has not been detected in clinical human tumors, its wild-type form has recently been implicated in mammalian malignancies, including neuroblastoma, melanoma, pancreatic, lung and breast cancers, and B-cell chronic lymphocytic leukemia. In addition, it was recently identified as a mutated gene in human cancers of various origins. However, the activity and contribution to cancer of these Vav1 mutants is still unclear. This review addresses the physiological function of wild-type Vav1 and its activity as an oncogene in human cancer. It also discusses the novel mutations identified in Vav1 in various cancers and their potential contribution to cancer development as oncogenes or tumor suppressor genes.

## INTRODUCTION

The past few decades have witnessed a major leap in understanding of the molecular mechanisms involved in tumor pathogenesis and progression [1]. Signaling molecules that play critical roles in cancer were identified and served as targets for therapeutic drugs. For instance, the first drug targeting a cancer gene, Herceptin, is helpful for many breast cancer patients with tumors driven by the target hormone receptor, HER2 [2]. Another example is the drug Gleevec, used for inhibition of protein kinase Abl in Chronic Myelogenous Leukemia (CML) patients [2]. Gleevec is also effective for treating Gastrointestinal Stromal Tumors (GIST) because it blocks the hormone receptor Kit, which often causes GIST [2]. Despite these significant advances, the pathogenic mechanisms of many signal transducer proteins implicated in cancers remain unknown.

One interesting signal transducer protein that is a potential target for cancer therapeutic drugs is Vav1. Vav1 was identified as an oncogene using the nude mouse tumorigenicity assay [3]. In this assay, NIH3T3 cells co- transfected with DNA from several esophageal carcinomas and the pSV2neo plasmid (which carries the gene that confers resistance to the G418 drug) were injected into nude mice [3]. These experiments led to the isolation of a novel human oncogene, which was designated Vav, the sixth letter of the Hebrew alphabet, because it was the sixth oncogene detected in Dr. Barbacid's laboratory [3]. The isolation of the Vav oncogene led to the identification of its wild-type (wt) form [4, 5] and subsequent identification of two additional mammalian members of this protein family, Vav2 [6] and Vav3 [7]. Nucleotide sequence analysis of the first Vav oncogene isolated, now termed Vav1, revealed that it was activated *in vitro* by replacement of 67 residues of its amino-terminus with sequences of pSV2neo, co-transfected as a selectable marker [3–5]. Although initially identified as an oncogene [3], Vav1 has been subsequently acknowledged as an important signal transducer with a pivotal role in the hematopoietic system, where it is exclusively expressed [8–13], as will be detailed below.

This review will focus on our recent understanding of the involvement of Vav1 in human cancer, the mechanism of ectopic Vav1 expression in cancer and its mode of function. The newly identified mutations in Vav1 in human cancer will be discussed.

### Structure

**Figure 1:**
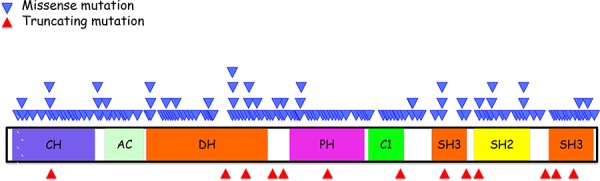
Schematic summary of Vav1 structure and location of various mutations identified in human cancers Vav1 encodes the following domains: calponin-homology (CH) domain; acidic (AC) motif, which contains 3 tyrosine residues; a DBL homology (DH) domain; a pleckstrin homology (PH) domain; a C1 domain; two SRC-homology 3 (SH3) domains; and a SRC-homology 2 (SH2) domain. The function of each region is detailed in the text. The location of missense mutations (light blue triangles) is indicated above the protein stricture and the location of truncations (red triangles) are depicted beneath. The information concerning these mutations is adapted from the catalogue of Somatic Mutations in Cancer (COSMIC) database.

Vav1 contains many characteristic structural motifs important for its function as a versatile signal transducer (Figure 1) [8–11]. These include: 1) a calponin-homology domain (CH; amino acids 3–121) which, in other proteins, associates with F-actin [14]. The Vav1 CH domain does not associate with F-actin, but is critical for Vav1's involvement in calcium mobilization [15]; 2) an acidic motif (AC; amino acids 133–193) that contains three regulatory tyrosines (Y142; Y160 and Y174) [16]; 3) a DBL homology (DH) region (amino acids 199–373), which exhibits a guanine nucleotide exchange (GEF) activity towards the Rho family GTPases [17]; 4) a Pleckstrin homology domain (PH) (amino acids 404– 505) that mediates interaction with phospholipids [18] resulting in Vav1 localization to the plasma membrane and regulation of Vav1 GEF activity [19]; 5) an atypical C1 (amino acids 515–564), which lacks the features required for lipid binding and instead might affect protein–protein interactions [20]; 6) a proline rich region (amino acids 606–610) that mediates binding of Vav proteins to Src homology 3 (SH3) containing proteins [21]; 7) a Src homology 2 (SH2) region (amino acids 672–746) that enables the binding of Vav1 to tyrosine phosphorylated proteins [22, 23]; 8) two SH3 domains (amino acids 615– 659 and 786–841) that mediate interactions with proline-rich domains [22, 23]; and 9) two nuclear localization signals (NLS; amino acids 487–494 and 576–589) [24]. Finally, Vav1 contains multiple tyrosine residues that affect its activity [25, 26].

### Biological functions of Vav 1

Vav1 participates in various cellular responses including actin cytoskeleton reorganization, gene transcription, and development and activation of immune cells. The role of Vav1 in the hematopoietic system has been extensively studied and reviewed [8–13] and therefore it will be only briefly summarized here.

The best-known function of Vav1 is its GEF activity for the Rho family of GTPases, an activity strictly dependent on tyrosine phosphorylation [7, 25, 27]. There have been conflicting reports on the substrate specificity of Vav1 [17, 27, 28], yet it is well accepted that Rac1 is the preferred substrate of Vav1 [17, 25, 27–29]. The nucleotide exchange activity of Vav1 on Cdc42, RhoA and RhoG is also enhanced, but to a lesser extent compared to Vav1's activity towards Rac [20].

In immune cells, endogenous Vav1 is tyrosine phosphorylated following activation of many receptors, including the T-cell receptor (TCR) [22, 23], B-cell receptor (BCR) [30], FcRI [31], cytokine receptors [32], NK receptors [33], chemokine receptors [34] and integrins [35]. The activation of Vav1 by these receptors leads to different outcomes depending on the specific hematopoietic cell type. For instance, Vav1 was shown to be associated with the formation of the immunological synapse (IS) in T cells [36, 37] and B cells [36] due to its activity as a regulator of cytoskeleton organization. Mice with Vav1-deficient T cells exhibited impaired cytoskeleton reorganization and impaired immune response [38, 39]. The GEF activity of Vav1 was also shown to be critical for activation of killing by Natural Killer (NK) cells [40]. In addition, Vav1^−/−^ mice are defective in their ability to eliminate tumors *in vivo* and in natural killing and antibody-dependent cellular cytotoxicity *in vitro* [41, 42]. In macrophages, genetic deletion of Vav1 shows that it is also required for Rac-dependent complement-mediated phagocytosis [43], cell migration [44] and macrophage chemotaxis to CSF-1 [45].

Vav1 also regulates the activity of multiple transcription factors in T cells in response to TCR stimulation, including Nuclear Factor of Activated T cells (NFAT), Activator Protein-1 (AP-1) and Nuclear Factor κB (NF-κB) [15, 46]. Vav1's ability to trigger release of calcium from inner reservoirs in T cells was found to be critical for this function [15].

Vav1 activates multiple signaling pathways, including the extracellular signal-regulated kinase (ERK) and c-Jun N-terminal kinase (JNK) pathways [25, 47, 48]. Murine Vav1-deficient T-cells exhibit defects in TCR-induced activation of ERK [15]. Moreover, Vav1 transduces TCR signals that lead to recruitment of the RasGEF, RasGRP1, Sos1 and Sos2 to LAT, leading to activation of ERK [49]. Also, over-expression of Vav1 and RasGRP1 in Jurkat T cells leads to hyperactivation of Ras [48].

Many of Vav1 functions are exerted via its ability to associate with other proteins [8–11]. For instance, several proteins associate with the CH region including Socs1, a downstream component of the Kit receptor tyrosine kinase signaling pathway [50]; ENX-1, a putative transcriptional regulator of homeobox gene expression [51]; Ly-GDI, a regulator of Rho GTPases [52]; and calmodulin [53]. The SH2 domain interacts with autophosphorylated tyrosine kinases such as ZAP-70 [54] and Syk [55] and with the adapter proteins: SLP76 [56] and Blnk [57]. Vav1's N-terminal SH3 domain binds the adapter protein Grb2 [58] shown to be necessary for translocation of Vav1 to the plasma membrane and its interaction with upstream tyrosine kinases in lymphoid cells [8]. The C-terminal SH3 domain of Vav1 forms complexes with a wide variety of proteins including cytoskeletal regulators (Zyxin) [59], RNA-binding proteins (hnRNP-K, hnRNP-C and Sam68) [60, 61], transcriptional modulators, ubiquitination factors, viral proteins, a Kruppel-like protein, and Dynamin 2 [8, 9, 62]. While the consequences of all these interactions are not yet known, Vav1's ability to interact with many proteins likely allows it to function in multiple signaling pathways, highlighting its involvement in multiple pathways.

Finally, Vav1 has an important role in hematopoietic cell development. T cells from Vav1-deficient mice demonstrated a partial block at the CD4−CD8^−^ (double negative; DN) to CD4^+^CD8^+^ (double positive; DP) transition and also at the transition from DP to CD4^+^CD8^−^ or CD4^−^CD8^+^ single positive (SP) [63]. Experiments performed with mice lacking Vav1 and the other members of the Vav protein family, i.e., Vav2 and Vav3, led to a 100-fold reduction in the number of DP and SP thymocytes and peripheral T cells, indicating that Vav2 and Vav3 proteins partially compensate Vav1 function in thymocyte development [64].

In summary, we know that Vav1 is a versatile signal transducer, critical for numerous biological activities in the hematopietic system, largely through its Rho-GEF activity. However, its interaction with numerous and diverse effectors suggests it plays additional roles in various signaling cascades.

## VAV1 REGULATION

Vav1 GEF activity is controlled through auto-inhibition of the DH region, which is conferred by a α-helix in the AC region. Tyr174 lies within this α-helix and directly binds the GTPase interaction pocket of the DH domain, blocking access to its substrate and inhibiting Vav1 GEF activity. Phosphorylation releases Tyr174 from the binding pocket, relieving the auto-inhibition [28, 65]. It has been suggested that the Vav1 CH domain can bind to the C1 region, also occluding the DH domain and blocking access to Rac/RhoGTPases. The CH-C1 interaction seemingly stabilizes the inhibitory Tyr174-DH interaction. Indeed, deletion of this domain results in constitutively active GEF activity [66]. In addition, the PH domain has been shown to regulate Vav1 catalytic activity by interaction with two lipid products of PI3K: phosphatidylinositol 4, 5-biphosphosphate (PIP2) and phosphatidylinositol 3, 4, 5-triphosphosphate (PIP3) [19, 67]. Whereas binding of PIP3 moderately enhances the *in vitro* GEF activity of Vav1, binding to PIP2 has an inhibitory effect. Recent high resolution X-ray structure of DH-PH-C1 domains suggested that PH and C1 domains contribute to GEF activity by stabilizing the DH domain structure and not through direct contacts with Rac/RhoGTPases [20].

### Vav 1 expression in human cancers

In the past decade numerous studies reported the unexpected expression of Vav1, which is usually found only in the hematopietic system, in a variety of human cancers. Ectopic Vav1 expression was first noted in the neuroblastoma SK-N-MC cell line [68]. The Vav1 protein in SK-N-MC exhibited the same molecular weight, phosphorylation state, and ability to bind to EGF receptor as wild-type Vav1 and had no mutations [68]. A subsequent screen of 42 primary human neuroblastoma tumors revealed that the majority (76%) expressed Vav1, suggesting for the first time that ectopic expression of wild type Vav1 might contribute to human cancer [68].

Vav1 was also identified in more than 50% of 95 pancreatic ductal adenocarcinoma (PDA) tumor specimens examined [69]. Patients with Vav1-positive tumors had a worse prognosis for survival compared to patients with Vav1-negative tumors [69]. Sequence analysis of the Vav1 cDNA from pancreatic cancer cell lines and tumors confirmed that they express intact wild-type Vav1 [69]. In addition, aberrant expression of Vav1 was found in 42% of 78 lung cancer cell lines examined, in 46% of 57 human primary lung cancer specimens [70] and in breast [71], ovarian and prostate cancers [26]. Grassilli et al., demonstrated that Vav1, a cytoplasmic expressed protein, is often found in the nucleus of early invasive breast tumors [72]. The high amounts of nuclear Vav1 in these tumors are positively correlated with low incidence of relapse [72]. Moreover, it was demonstrated that when it is expressed in the nucleus it can modulate genes that are associated with the metastatic process [72]. Several melanoma cell lines also express wild-type Vav1, including the highly metastatic BLM cells, although the level of protein expression was low and it was localized in the cell periphery near the plasma membrane [73]. Finally, a large screen of medulloblastomas identified widespread expression of Vav1 in the majority of specimens analyzed and Vav1 was demonstrated to play a critical role in medulloblastoma tumor maintenance, with Vav1 abrogation markedly reducing medulloblastoma growth [74].

Intriguingly, and perhaps counter-intuitively, Vav1 does not appear to be significantly involved in hematological malignancies [75]; however, from work reported thus far, this does not seem to be the case. Prieto-Sanchez *et al*. [76] examined Vav1 protein levels and phosphorylation status in 118 unselected cases of hematologic neoplasms. They found that Vav1 was phosphorylated and overexpressed in 10 of 14 cases of B-CLL with 13q deletion, however no change in its level of expression was recoreded in any of the myeloproliferative neoplasms examined [76]. Bertagnolo *et al*. have demonstrated in a series of studies that Vav1 is required for the retinoic ATRA-induced differentiation of human promyelocytic leukemia cell lines to neutrophils as well as PMA-induced maturation of these same cell lines to monocytes/macrophages [77, 78]. However, there is no evidence as to whether Vav1 has a role in this specific tumor development.

Thus, the accumulating data clearly point to an important role of ectopically expressed wild-type Vav1 in human cancer [24].

## WHY IS VAV1 EXPRESSED IN CANCER?

Neither the physiological nor pathological regulation of Vav1 expression is completely understood. One mechanism suggested to play a role in ectopic expression of Vav1 in cancer of non-hematopoietic origin is the methylation status of the Vav1 promoter. Bisulfite sequencing revealed that the Vav1 promoter was completely unmethylated in human lymphocytes, but methylated to various degrees in healthy tissues that do not normally express Vav1 [79]. Fernandez-Zapico, *et al*., demonstrated that epigenetic changes in the Vav1 gene, but not gene amplification, contributed to its aberrant expression in pancreatic cancer cell lines [69]. These results are further substantiated by a recent report indicating that Vav1 was identified by cross-species epigenetics to play a critical role in maintenance of Sonic Hedgehog (SHH) subgroup medulloblastoma tumors (MBSHH) [74]. This study identified widespread hypo-methylation of Vav1, leading to its elevated expression, as a conserved aberrant epigenetic event that characterizes the majority of MBSHH tumors and is associated with poor outcome in MBSHH patients. These findings establish Vav1 as an epigenetically regulated oncogene with a key role in MBSHH maintenance [74].

Another mechanism emerged from our studies aimed at identifying transcription factors that regulate Vav1 expression [79]. We demonstrated that mutations in putative transcription factor binding sites at the Vav1 promoter affect its transcription in cells of different histological origin [79]. Among these sites is a consensus site for c-Myb, a hematopoietic-specific transcription factor also found in Vav1-expressing lung cancer cell lines. Depletion of c-Myb using siRNA led to a dramatic reduction in Vav1 expression in these cells [79]. Consistent with this, co-transfection of c-Myb activated transcription of a Vav1 promoter-luciferase reporter gene construct in lung cancer cells devoid of Vav1 expression. Together, these results indicate that c-Myb is involved in Vav1 expression in lung cancer cells. The possibility that additional transcription factors play a role in Vav1 expression in cancer cells remains to be explored.

## ROLE OF VAV1 IN HUMAN CANCER

Vav1 functions physiologically in numerous pathways, therefore it is somewhat difficult to attribute its multiple activities in cancer to a particular pathway. Nonetheless, the main role attributed to Vav1 in cancer is its activity as a GEF for Rho/RacGTPases [9]. The Rho/RacGTPases function as molecular switches in a variety of signaling pathways following stimulation of cell surface receptors. Rho/RacGTPases regulate numerous cellular processes that become dysregulated in cancer, including cytoskeleton organization, gene transcription, cell proliferation, migration, growth and survival [13, 80]. It therefore seems reasonable that defects in Rho/RacGTPase pathway regulation may be involved in the development of cancer [13, 81]. Consistent with this, various GEFs have been implicated recently in cancer [13, 26], and activation of Vav1 GEF activity following tyrosine phosphorylation has been demonstrated in EGF and PDGF stimulated NIH3T3 fibroblasts expressing Vav1 [22, 23] as well as in cancer cells, including neuroblastoma [68], pancreatic cancer [69] and lung cancer [70]. It is also activated following stimulation of CSF1R in lung cancer cells [82]. The truncated Vav1 oncogene first identified as an oncogene exhibited constitutive activity as a GEF [25]. A mutation at tyrosine 174, leading to enhanced GEF activity, results in increased transformation [83], further highlighting the importance of the GEF activity of Vav1 for transformation. Also, Fernandez-Zapico *et al*., demonstrated that, unlike wild-type Vav1, a GEF-defective Vav1 mutant cannot restore proliferation of pancreatic cancer cells depleted of Vav1 [69]. Interestingly, Vav1 expression was required for proliferation even in the presence of mutant K-Ras in pancreatic and lung cancer, demonstrating the critical role of Vav1 in tumor development [69, 70]. Razidlo *et al*. recently reported that Vav1 is required for Rac1-mediated formation of lamellipodia and subsequent migration of tumor cells [84]. In addition, Vav1 is a potent regulator of transendothelial migration of leukocytes, and also contributes to CXCL12-induced MT1-MMP expression and invasion by melanoma cells [73]. Additionally, Razidlo *et al*., demonstrated that Vav1 is involved in invasion and migration through the formation of invadopodia and matrix degradation [85]. This process requires Vav1 activation of Cdc42, demonstrating that in pancreatic tumor cells, ectopically expressed Vav1 can signal through multiple pathways. This is consistent with a previous report that Vav1-induced oncogenic transformation requires multiple signaling pathways, including Rac1, Cdc42, and RhoA, as well as NFκB and JNK [86]. Together, the above findings imply that GEF activity is critical for Vav1's role in cancer cell migration and invasion and suggest that ectopically expressed Vav1 acts as an upstream activator of Rac1, RhoA and possibly Cdc42 signaling pathways in response to extracellular stimulation, leading to cytoskeleton changes and ultimately to increased cell motility.

Activation of Vav1 also stimulates MAPK signaling cascades, which may contribute to cancer by enhancing cell mitogenic properties. MAPK function is related to multiple biologic processes such as cell proliferation, differentiation, death, migration, invasion and inflammation [87]. It is well established that once MAPK is abnormally activated, cancer may occur [87]. Numerous studies have described Vav1's physiological role in ERK signaling in the immune system [8]. Since Rho GTPases were reported to control cytoskeleton organization and cellular activities, such as the JNK (c-Jun N-terminal kinase) and p38 MAPK (mitogen-activated protein kinase) cascades [88], it is conceivable that Vav1 also controls these pathways in cancer. Indeed, we recently demonstrated that ERK phosphorylation is dependent on Vav1 activation in lung cancer cells [82].

Our recent data suggest that Vav1 may also contribute to cancers by regulating growth factor expression. We found that lung cancer cells depleted of Vav1 exhibit significantly reduced levels of the hematopoietic growth factor CSF1, suggesting that Vav1 propagates an autocrine feed forward loop by upregulating expression of growth factors [82]. Transcriptome analysis demonstrated that Vav1 depletion results in a marked reduction in CSF-1 expression. The association between Vav1 expression and CSF1 was further supported by signal transduction experiments, pointing to the involvement of Vav1 in regulating the lung cancer secretome [82]. Blocking ERK phosphorylation led to a decrease in CSF1 transcription, suggesting a role for ERK, a downstream effector of Vav1, in CSF1 expression [82]. CSF1-silenced cells exhibited reduced focus formation, proliferation abilities, and growth in *NOD/SCID* mice. CSF1-silenced H358 cells resulted in significantly smaller tumors, showing increased fibrosis and a decrease in tumor infiltrating macrophages. Finally, immunohistochemical analysis of primary human lung tumors revealed a positive correlation between Vav1 and CSF1 expression, which was associated with tumor grade [82]. Our results suggest a potential cross-talk between cancer cells and the microenvironment controlled by CSF1/Vav1 signaling pathways. This indicates that Vav1 might be involved in additional pro-tumorigenic pathways in addition to its GEF activity. It is noteworthy that lung cancer cells depleted of Vav1 also showed a decrease in EGF [82] and TGFα [70], further highlighting the association between Vav1 expression in cancer cells and the expression of autocrine/paracrine growth factors.

The possibility that Vav1 can stimulate secretion of autocrine ligands was also suggested for the human mammary epithelial cell line MCF-10A, in which expression of a constitutively active form of Vav1 promoted migration and morphological changes [89]. This increased migration was dependent on Vav1 GEF activity, which stimulated the Rac1–Pak pathway, and also on secretion of an autocrine EGF receptor ligand. We previously reported that the secretion of osteopontin, a CD44 and integrin ligand known to be associated with invasion, progression and metastasis, is upregulated by oncogenic Vav1 in NIH3T3 cells [90]. These data support the existence of feed-forward loops in which Vav1 regulates secretion of autocrine ligands leading to receptor stimulation and subsequent increases in Vav1 activation. The expression and function of many other proteins appear to be affected by Vav1 [82], yet the exact contribution of such proteins for Vav1-dependent tumorigenicity has not been explored.

Vav1 might also contribute to transformation by influencing cell cycle progression and gene transcription. Indeed, as shown in pancreatic cancer cells, EGF stimulation leads to tyrosine phosphorylation of Vav1, followed by the activation of a Rac1/Pak1/NF-κB signaling pathway resulting in an increase in cyclin D1 which leads to enhanced pancreatic tumor cell proliferation [69]. This recurring theme suggests that Vav1 might contribute to the progression of cancer by regulating secretion of autocrine ligands critical for tumorigenicity, as well as affecting the expression of other proteins critical for various cellular functions.

The ability of Vav1 to contribute to cancer development was recently also attributed to its expression in cells of the microenvironment. Garcia JL *et al*., demonstrated by immunohistochemical survey of 59 high-grade gliomas that Vav1 is found in non-tumoural astrocyte-like cells in peri-tumor or peri-vascular locations, but not in the glioblastoma cells [91]. Thus, in this case, expression of Vav1 is linked to synergistic signaling cross-talk between cancer cells and infiltrating cells, a phenomenon that could have a role in the neoplastic process in glioblastoma tumors [91].

In summary, several of Vav1's intracellular mechanisms can contribute to tumorigenicity, including activation of RhoGTPases, activation of cyclin D1 and NF-κB, and protein-protein interactions. Further research is required to provide more definitive insight into mechanisms underlying Vav1's role in human cancer.

## MUTATIONS IN VAV1 IN HUMAN CANCER

**Figure 2:**
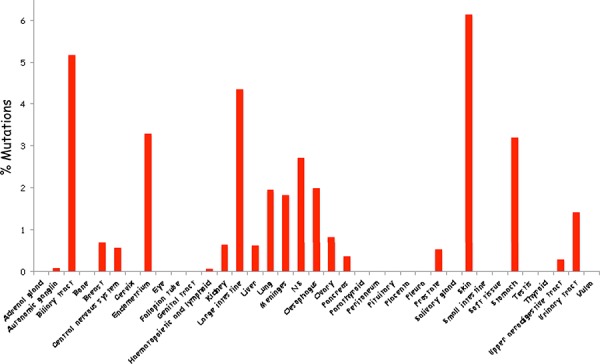
Schematic summary of Vav1 mutations in cancer of various tissue origins The percentage of Vav1 mutations in numerous tissues was calculated according to the information available from the catalogue of Somatic Mutations in Cancer (COSMIC) database.

While evidence over the last decade substantiated Vav1 overexpression in human cancer, the question remained whether mutations in Vav1 contribute to human cancers [68–71, 73, 74, 76]. Based on data recently obtained from human genome sequencing coordinated by the Wellcome Trust Sanger Institute, Vav1 appears to be mutated in ∼1% of human cancer of multiple tissue origins (Figure 1, http://cancer.sanger.ac.uk/cosmic/gene/analysis?ln=Vav1&ln1=CBL&start=1&end=907&coords=AA%3AAA&sn=&ss=&hn=&sh=&id=5003#). Some cancers exhibit a higher occurrence of Vav1 mutations, such as those originating in the biliary tract (5.17%), endometrium (3.23%), large intestine (4.35%), and skin (6.13%), possibly attesting to the importance of the molecular lesions in Vav1 in certain tissues (Figure 2). Since the isolation of Vav1 numerous studies have attempted to decipher its structure/function by introducing mutations at different domains. Therefore, it is interesting to compare the mutations found in human cancer to those experimental mutations. The mutations identified in Vav1 in human cancer span all its cardinal domains. CH Region: Mutations are at residues that are outside the backbone of this domain and are conserved in more than 62% of the sequences [92]. Several of these mutations (L17V, E59K, E84D, L88F, E95K, and W117R) are at isoleucine, leucine, valine, phenylalanine and tryptophan residues frequently found in this region, suggesting these residues may be involved in transformation. DH Region: Human cancer-associated mutations occur in highly conserved residues, including E201K, L322M, D324Y, H337Y, L339I, T347M, V373F and E378K [93]. An additional mutant, Y283F, is not conserved among all the proteins, but is shared with Dbl. PH Region: The only conserved residue within the PH region that has been shown to be mutated in human cancers is E408K [93]. C1 Region: Interestingly, several of the residues mutated in human cancers (Q542E, R548L, E556K and P562S), were previously mutated experimentally by Zugaza *et al*., [66] but with different amino-acid substitutions. Since Zugaza *et al*., substituted with alanine [66], the activity of the naturally occurring mutants may differ from Zuzaga's results. SH2 Domain. Several Vav1 mutations in cancer occur at highly conserved residues in the SH2 domain, including R678Q, E682E, G691R, and R696W.

For a long period it was believed that truncation of the amino-terminus was the only molecular lesion that converted wild-type Vav1 to a transforming gene in NIH3T3 fibroblasts [4, 5]. The importance of the amino-terminus for Vav1 activity as a transforming gene was subsequently attributed to tyrosine 174. Thus, mutation at tyrosine 174 greatly enhances the transforming activity of Vav1 [83], since it relieves the autoinhibition of GEF activity by the acidic region [28]. One additional mechanism for Vav1 activation was recently reported by Razanadrakoto et al., [94] demonstrating that a mutation at D797 (D797N) in the carboxy SH3 region of wild-type Vav1 endowed the protein with transforming properties [94]. The same mutation introduced in the Vav1 oncogene did not change its transforming potential, suggesting that they operate in a similar manner [95]. Although numerous mutations have been experimentally introduced in Vav1 throughout the years, including in the DH, PH, C1, both SH3 regions and the SH2, none led to increased transformation of Vav1-expressing NIH3T3 fibroblasts [26, 66, 95, 96], except the ones mentioned above. Therefore, it remains to be tested whether the cancer-identified Vav1 mutants are transforimg in such experimental conditions.

The pattern of mutations in Vav1 is puzzling. Both missense mutations and protein-truncating alterations are found throughout the Vav1 protein (Figure 1). Vogelstein and colleagues concluded that such a pattern of mutations is typical for tumor suppressor genes, while oncogenes are recurrently mutated at the same amino acid positions [97]. However, in view of the extreme functional complexity of the Vav1 protein, we cannot draw the same conclusion for Vav1 without further study. It is possible that aberrant function of each of the different domains of Vav1 can have different consequences in different cell types, or different pathophysiological processes, leading independently to transformation.

Several examples support the idea that genes can have dual roles as oncogenes and tumor suppressor genes depending on the specific mutation and tissue distribution. p53, a well-known tumor suppressor gene, can also function as an oncogene when it carries a gain-of-function mutation [98]. Thus, some mutant p53 proteins gain oncogenic functions through which they actively contribute to establishment, maintenance and spreading of cancer cells [98]. Also, some functional studies suggest that NOTCH1 is an oncogene, whereas others suggest it is a tumor suppressor gene [99]. In hematological malignancies such as lymphomas and leukemias, NOTCH1 mutations were often recurrent and did not truncate the predicted protein [100], while in certain solid tumors, the mutations were not recurrent and were usually inactivating [101]. Thus different mutations to the same protein (NOTCH1) lead to its involvement in different tumor types through distinct mechanisms.

We recently demonstrated that Vav1 plays a dual role as a pro- or an anti-apoptotic protein in breast cancer cells, depending on whether the cells express p53 [71]. p53 is required for the pro-apoptotic effect of Vav1 in these breast cancer cell lines [71]. Whether these experiments point to a possible dual role of Vav1 in cancer, depending on the specific mutation and the specific cell-type, remains to be carefully studied. In light of the fact that Vav1 is mutated in just 1% of 20427 cancer specimen analyzed, and in view of its complex biochemical structure and diverse cellular functions, it is prudent to await the identification of a larger number of mutations before we draw conclusions about the true identity of Vav1 mutants (oncogene/tumor suppressor gene), as suggested by Lawrence et al., who explored the feasibility of creating a comprehensive catalogue of cancer genes [102].

## ROLE OF VAV2 AND VAV3 IN HUMAN CANCER

Whereas the expression of Vav1 appears to be predominantly limited to the hematopoietic system, Vav2 and Vav3 are expressed more ubiquitously [3, 6, 7]. The various members of the Vav family of proteins (Vav1, Vav2 and Vav3) exhibit redundant as well as distinct functions in development [64, 103]. Both Vav2 and Vav3 have been implicated in cancer. High expression of Vav2 is implicated in cancers such as oral squamous cell carcinoma [104], squamous carcinomas of the head and neck [105], and prostate cancer [106]. Also, high levels of Vav3 have been observed in various types of cancers, including glioblastoma [107], prostate cancer [108] and colorectal cancer [109]. Vav3 was also shown to be significantly upregulated in breast cancers compared with benign breast diseases [110, 111]. Furthermore, Vav3 was identified as a biomarker of a poor prognosis in breast and ovarian cancers [112, 113]. Like Vav1, Vav2 and Vav3 become oncogenic following N-terminal truncation [7, 27], yet there appears to be Vav isoform-distinct functions in cancer. For instance, specific depletion of only Vav1 in pancreatic cancer cell lines led to inhibition of their growth, despite the continuous expression of Vav2 [69]. Moreover, knockdown of Vav2 in these cells did not alter their growth [69], suggesting that Vav1 and Vav2 play different roles in pancreatic cancer cells. Contrary, depletion of Vav2 and Vav3 could result in a dramatic effect on tumor growth of a different histological origin. Thus, reduction of Vav2 and Vav3 expression in mouse mammary tumor cells led to a decline in metastatic growth, similar to the effect of Vav1 depletion in pancreatic cancer cells [114]. These results further highlight the fact that the various members of the Vav family of proteins, Vav1, Vav2 and Vav3, may have different roles in human cancer.

## CONCLUDING REMARKS

Vav1 is a signal transducer protein that functions exclusively in the hematopoietic system under normal physiological conditions. It participates in signal transduction events through tyrosine phosphorylation-dependent guanine nucleotide exchange activity. While Vav1 was first identified as an oncogene capable of inducing transformation in NIH3T3 fibroblasts, accumulating results from the past decade clearly indicate its participation in human cancer through ectopic overexpression. Research on Vav1 in recent years has cycled between furthering our understanding of its physiological function in the hematopoietic system and studying its involvement in human malignancies. While it is now clear that Vav1 expression is deregulated in some cancers, leading to expression outside the hematopoietic system, it also emerges now as a mutated gene in human cancers of various origins; however, the activity and contribution of the various mutations is still unclear. The biological importance of the Vav1 mutants identified in human cancer needs further exploration, including testing the role of the various mutants in cognate tissues, assessing GEF activity of mutants, and testing their ability to associate with other proteins. Despite these many open questions, the existing data suggest Vav1 as a promising target for drug design, especially blocking its GEF activity, as was recently implied by Razidlo *et al*., who demonstrated that inhibition of Vav1 by drugs leads to inhibition of pancreatic cancer metastasis [115].
